# AI system for diagnosing mucosa-associated lymphoid tissue lymphoma and diffuse large B cell lymphoma using ImageNet and hematoxylin and eosin–stained specimens

**DOI:** 10.1093/pnasnexus/pgaf137

**Published:** 2025-04-30

**Authors:** Shuto Yamaguchi, Teijiro Isokawa, Nobuyuki Matsui, Naotake Kamiura, Tatsuaki Tsuruyama

**Affiliations:** Department of Electronics and Computer Science, Graduate School of Engineering, University of Hyogo, Himeji 671-2201, Japan; Department of Electronics and Computer Science, Graduate School of Engineering, University of Hyogo, Himeji 671-2201, Japan; Department of Electronics and Computer Science, Graduate School of Engineering, University of Hyogo, Himeji 671-2201, Japan; Department of Electronics and Computer Science, Graduate School of Engineering, University of Hyogo, Himeji 671-2201, Japan; Department of Drug Discovery Medicine, Graduate School of Medicine, Kyoto University, Kyoto 606-8315, Japan; Department of Clinical Laboratory, Graduate School of Health Sciences, Kyoto Tachibana University, Kyoto 607-8175, Japan

**Keywords:** convolutional neural networks, pathological diagnosis, malignant lymphoma

## Abstract

AI-assisted morphological analysis using whole-slide images (WSIs) shows promise in supporting complex pathological diagnosis. However, the implementation in clinical settings is costly and demands extensive data storage. This study aimed to develop a compact, practical classification model using patch images selected by pathologists from representative disease areas under a microscope. To evaluate the limits of classification performance, we applied multiple pretraining strategies and convolutional neural networks (CNNs) specifically for the diagnosis of particularly challenging malignant lymphomas and their subtypes. The EfficientNet CNN, pretrained with ImageNet, exhibited the highest classification performance among the tested models. Our model achieved notable accuracy in a four-class classification (normal lymph node and three B cell lymphoma subtypes) using only hematoxylin and eosin-stained specimens (AUC = 0.87), comparable to results from immunohistochemical and genetic analyses. This finding suggests that the proposed model enables pathologists to independently prepare image data and easily access the algorithm and enhances diagnostic reliability while significantly reducing costs and time for additional tests, offering a practical and efficient diagnostic support tool for general medical facilities.

**Significance Statement**

AI can improve pathologic diagnostic efficiency, but existing systems often require large datasets and advanced computational resources, limiting accessibility and raising concerns about patient trust. This study introduces a simple, cost-effective AI model that pathologists can fine-tune using images selected during routine microscopic examination. Our model achieved diagnostic accuracy comparable to advanced techniques such as genetic testing and classified challenging cases, including malignant lymphoma, with minimal resources. This approach enables efficient diagnostics in resource-limited settings while maintaining patient trust. By demonstrating an accessible and reliable AI framework, this study offers a practical pathway for integrating AI as a supportive tool in clinical practice, advancing the role of AI in enhancing medical diagnostics.

## Introduction

AI has significantly advanced early disease detection and improved diagnostic accuracy by analyzing medical images such as X-rays, CT scans, and MRIs, thereby helping to mitigate radiologist shortages (1). Additionally, research is progressing in optimizing surgical procedures through AI analysis of large video datasets (2, 3). Pathology presents unique challenges owing to its high-resolution, information-rich nature (4). Moreover, the lack of standardization in the preparation of microscope specimens, which serve as the basis for imaging, poses an additional challenge (5).

In recent years, a large number of whole-slide images (WSIs) captured by scanners are stored as digital images. Many systems have been developed to analyze WSIs using digital image analysis based on machine learning algorithms to support diagnostics (6). Machine learning techniques commonly used in digital pathology image analysis can be divided into supervised and unsupervised learning. The purpose of supervised learning is to use training data to infer a function that can properly map an input image to an appropriate label (e.g. cancer). The label is associated with the WSI or an object within the WSI. Supervised learning algorithms include support vector machines, random forests, and deep learning. In particular, convolutional neural networks (CNNs) in deep learning are used to optimize features and classifiers simultaneously, and features learned by CNNs often outperform other traditional features in histopathological image analysis. However, implementing AI using WSI in digital pathology presents several significant challenges, including implementation costs, data management, and standardization of data formats. There are also fundamental issues, such as how physicians should explain AI-driven diagnostics to patients and whether the AI diagnoses are trustworthy. Despite these challenges, addressing the barriers to AI-assisted diagnostics is essential, considering the potential benefits of improving patient outcomes. These benefits include faster diagnosis through prediagnostic image screening (7, 8).

In this study, we evaluated the cancer classification performance of several convolutional CNN models with established accuracy, utilizing compact pathological images that are easy for pathologists to manage. The efficacy of such approaches was tested in diagnosing malignant lymphomas, which are often challenging for pathologists to diagnose based solely on morphological observation. The diagnosis of lymphomas frequently requires supplementary data from costly and time-intensive genetic analyses or immunophenotypic assessments (9).

First, we selected malignant lymphomas as examples, because their diagnosis requires complex and costly pathological techniques, including genetic analysis and immunohistochemistry (IHC) staining. This is a tissue staining technique that uses special dyes to detect specific proteins in tissues, helping pathologists understand diseases such as cancer. The results take ∼1 week to be returned, which is comparable to the time required for genetic testing. The samples include extranodal marginal zone lymphoma of mucosa-associated lymphoid tissue (MALT lymphoma (10)), a low-grade malignancy linked to lymphocytic infiltration, which can progress to diffuse large B cell lymphoma (DLBCL), a high-grade malignancy. Additionally, we aim to subclassify DLBCL based on the cell of origin—the germinal center B cell type (GCB) vs. non-GCB type (or activated B-cell-like lymphoma) (11–15). The non-GCB type has resulted in shorter malignant disease-free and overall survival rates (16–19), as well as a low response to nivolumab (20), highlighting the clinical importance of distinguishing these subtypes. Therefore, this differentiation is clinically significant.

The current study challenges to develop a compact model to categorize pathological images into normal vs. lymphoma, or into lymphoma subtypes such as MALT lymphoma, DLBCL GCB type, and DLBCL non-GCB type, for two-, three-, and four-class classification tasks. Pretraining datasets, such as the Columbia-Utrecht Reflectance and Texture Database (CUReT) (21), ImageNet (22), or a combination of both that can tuned by pathologists, are used for pretraining. CUReT and ImageNet are image datasets that are used for training and evaluating machine learning models. CUReT is a dataset specialized in the properties of textures and materials, whereas ImageNet is a general-purpose dataset broadly applicable to object recognition. Classification models using a set of CNNs, including AlexNet (23), VGG16 (24), ResNet18 (25), SqueezeNet (26), GoogleNet (27), and EfficientNet (28), are examined and compared to determine the most effective classifier combinations. Each model varies in complexity, efficiency, and accuracy, offering diverse strengths for classifier evaluation. In particular, EfficientNet is a compact, efficient CNN architecture that balances performance and computational cost, making it ideal for mobile applications. If the classification of diagnostically challenging lymphomas is successfully achieved, the utility of the compact model will be validated, leading to the realization of AI implementation as a diagnostic support tool in clinical settings.

## Results

### Deep learning of images

**Fig. 1.**
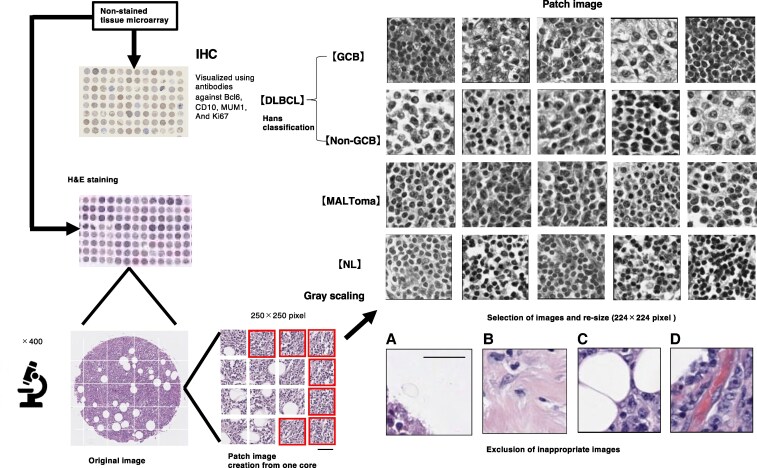
Process of providing training image data to the AI model. TMAs for DLBCL were stained using H&E, and for BCL6, MUM1, and CD10, IHC was used to acquire essential data for Hans’ classification. Based on the positivity or negativity of these stains, the samples were labeled as either GCB or non-GCB. Each image was captured at 400× magnification. The original images were divided into 16 patch images. From these, patches that lacked sufficient tissue content (A), fibrotic scar tissue (B), adipose tissue (C), or blood vessels (D) were excluded. The remaining images were resized and provided as teacher image data to the model. Scale bars represent 20 µm.

**Fig. 2.**
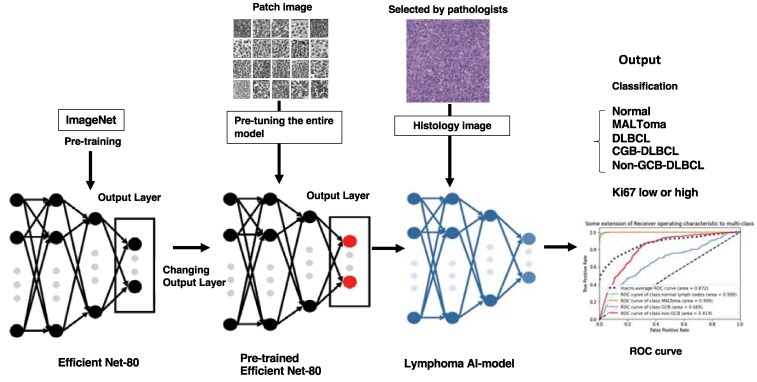
Construction process of the lymphoma AI classification model. The model was developed based on EfficientNet-B0, which was pretrained using ImageNet. Images selected by pathologists from tissue cores, typical images obtained in the final process outlined in Fig. 1, were used to create the lymphoma AI classification model. After model construction, the outputs included the differentiation between NL, MALT lymphoma (extranodal marginal zone lymphoma of mucosa-associated lymphoid tract lymphoma), GCB-type DLBCL, non-GCB-type DLBCL, and the risk related to Ki67 (either high or low).

This study analyzed data from 160 patients, comprising 25 normal lymph nodes (NL), 26 MALT lymphoma, 31 GCB, and 78 non-GCB cases purchased from Biomax tissue microarrays (TMAs) (Fig. 1 and Table 1). The details about the patients and anatomic sites from which the tissues were sourced, as well as the number of training and test data samples, are given in Table S1. The samples were initially stained with H&E staining for microscopic observation. After confirmation by two hematopathologists, all original images were preprocessed as follows: (i) each original image was divided into 16 parts to create 250 × 250 pixel images; (ii) images with an average RGB pixel value exceeding 210, which indicated an insufficient sample tissue imaging area, were excluded; (iii) images containing features unsuitable for training the identification model, such as tissue edges (Fig. 1A), scar fibrosis (Fig. 1B), adipose tissue (Fig. 1C), and blood vessels (Fig. 1D), were also removed; (iv) to mitigate variations in staining intensity among samples, grayscale conversion (output channels = 3) was applied to all patch images; and (v) all images were resized to 224 × 224 pixels to match the recommended input size of EfficientNet (Fig. 1: image after processing). According to Hans’ criteria, cases are classified using IHC as the GCB subtype if lymphoma cells were CD10^+^ or CD10^−^ Bcl6^+^ MUM^−^. Conversely, cases are classified as the non-GCB subtype if lymphoma cells were CD10^−^ Bcl6^−^ or CD10^−^ Bcl6 ^+^ MUM1^+^. The process of constructing the lymphoma AI classification model is illustrated in Fig. 2.

**Table 1.** Profiling of patients with MALT lymphoma, DLBCL GCB subtype, and DLBCL non-GCB subtype.

MALT lymphoma				
Sex	Female	9	Male	17
Age (years)	<20	3	20–39	5
	40–60	14	60<	4
Anatomic site	Parotid gland	2	Stomach	6
	Liver	2	Small intestine	3
	Colon	13		
			Total	26
GCB DLBCL				
Sex	Female	6	Male	25
				
Age (years)	<20	2	20–39	12
	40–60	13	60<	4
Phenotype	CD10^+^	28	CD10^−^ Bcl6 ^+^ MUM1^−^	3
Anatomic site	Abdominal cavity	2	Colon	4
	Groin	4	Neck	3
	Lymph node	1	Mesentery	2
	Retroperitoneum	3	Pelvic cavity	1
	Shoulder	1	Small intestine	4
	Stomach	6		
			Total	31
Non-GCB DLBCL				
Sex	Female	35	Male	43
				
Age	<20	3	20–39	21
	40–60	42	60<	12
Phenotype	CD10^−^ Bcl6^−^	5	CD10^−^ Bcl6 ^+^ MUM1^+^	73
Anatomic site	Abdominal cavity	1	Armpit	7
	Brain	1	Broad ligament	1
	Colon	13	Gallbladder	1
	Groin	9	Ischium	1
	Liver	1	Lumbar part	1
	Mediastinum	4	Mesentery	1
	Neck	19	Omentum	3
	Parotid gland	1	Pelvic cavity	2
	Retroperitoneum	5	Shoulder	2
	Stomach	1	Submaxillary	2
	Thyroid gland	1	Tongue	1
			Total	78
NL				
Sex	Female	4	Male	21
				
Age (years)	<20	0	20–39	14
	40–60	11	60<	0
			Total	25

### AI classification models

**Fig. 3.**
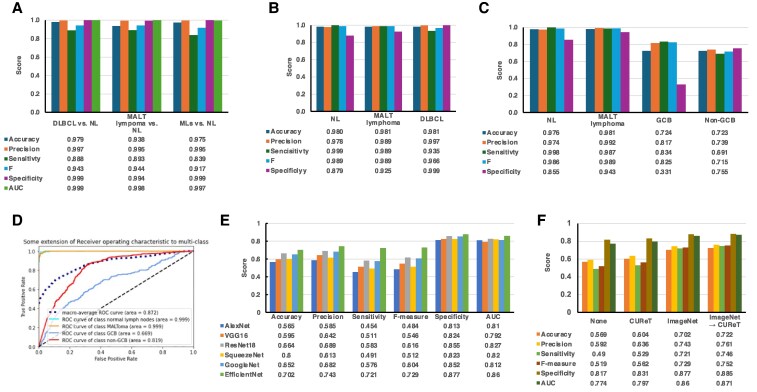
Model performance evaluation. A) Evaluation metrics for the two-class classification models: DLBCL vs. NL; MALT lymphoma vs. NL; and all lymphomas (MLs) vs. NL. B) Evaluation metrics for three-class classification models. C) Evaluation metrics for classifications among NL, MALT lymphoma, GCB DLBCL (GCB), and non-GCB DLBCL (non-GCB). D) Examples of ROC curves. E) Evaluation metrics for four-class classification among NL, MALT lymphoma, GCB, and non-GCB using AlexNet, VGG16, ResNet18, SqueezeNet, GoogleNet, and EfficientNet. F) Evaluation metrics for four-class classification among NL, MALT lymphoma, GCB, and non-GCB using no pretraining, pretrained with CUReT, pretrained with ImageNet, and pretrained with ImageNet followed by CUReT.

We developed a two-class model to distinguish NL from B cell lymphomas (Fig. 3A), a three-class model to differentiate NL, MALT lymphoma, and DLBCL (Fig. 3B), and a four-class model to classify NL, MALT lymphoma, GCB, and non-GCB (Fig. 3C).

In the two-class model, for case 1 (NL vs. DLBCL), the specificity was 0.89, while other evaluation metrics (accuracy, precision, sensitivity, and *F*-value) exceeded 0.94, indicating high performance. In case 2 (NL vs. MALT lymphoma), all metrics exceeded 0.89, demonstrating the effectiveness of the proposed method. In case 3 (NL vs. all lymphomas), the specificity stood at 0.84, but the other metrics again exceeded 0.92 (Fig. 3A). These findings underscore the robustness of our model, proving its ability to reliably distinguish NL from lymphomas. An example of the Receiver Operating Characteristic (ROC) curve and the confusion matrix in each case is shown in Fig. S1.

Next, we attempted to classify NL vs. MALT, NL vs. DLBCL, and MALT vs. DLBCL. In the created three-classification model, all evaluation metrics surpassed the 0.900 mark except for the specificity in classifying NL, which was 0.88 (Fig. 3B). Classification performance was assessed as binary classifications for each category against the remaining two classes. The average AUC for the three-class classification reached 0.999. These results indicate that the model is highly effective for clinical diagnostic support and is capable of accurately classifying NL, MALT lymphoma, and DLBCL from H&E-stained images. An example of an ROC curve and a confusion matrix is shown in Fig. S2.

In the four-classification model: NL, MALT lymphoma, and DLBCL, the AUC reached 0.87 in one test, with an average of 0.86 across 10 tests (Fig. 3C and D). Specifically, in the creation of this model, to achieve optimal classification performance, this study employed five CNN architectures: AlexNet, VGG16, ResNet18, SqueezeNet, GoogleNet, and EfficientNet, to compare the four-class classification performance. The results showed that in terms of AUC values, the performance ranking was as follows: 0.81, 0.79, 0.83, 0.82, 0.81, and 0.86, with EfficientNet demonstrating the most superior performance (Figs. 3E and S3A-F). Thus, EfficientNet emerged as the most effective model in terms of classification performance (28). Notably, EfficientNet demonstrated particularly high classification performance for MALT lymphoma, surpassing the other CNN models.

Furthermore, for pretraining EfficientNet-B0—the smallest model in the EfficientNet series, known for its lightweight structure and low computational cost—we evaluated its classification performance using the CUReT database. The experimentation involved four pretraining database combinations: no pretraining (Fig. S4A), CUReT alone (Fig. S4B), ImageNet alone (Fig. S4C), and both ImageNet and CUReT (Fig. S4D**)**. To evaluate the effect of pretraining, we further validated our four-class classifier using different pretraining scenarios: no pretraining, using only CUReT, using only ImageNet, and using both CUReT and ImageNet. The results showed that pretraining with ImageNet alone significantly enhanced classification performance (Fig. 3F). While combining both ImageNet and CUReT yielded some metric improvements, the overall enhancement was not substantial (Fig. S4D). Therefore, pretraining with ImageNet alone is sufficient, highlighting the potential of our compact model for identifying different types of B cell lymphomas. Thus, the development of an AI diagnostic system capable of simultaneously classifying lymphatic tissues into challenging categories such as MALT lymphoma and DLBCL (GCB type and non-GCB type) represents a significant advancement. This system is sufficient as a supportive tool for lymphoma diagnosis. The clinical significance of this achievement is detailed in the Discussion section.

### Visualization of rationale using gradient-weighted class activation mapping

**Fig. 4.**
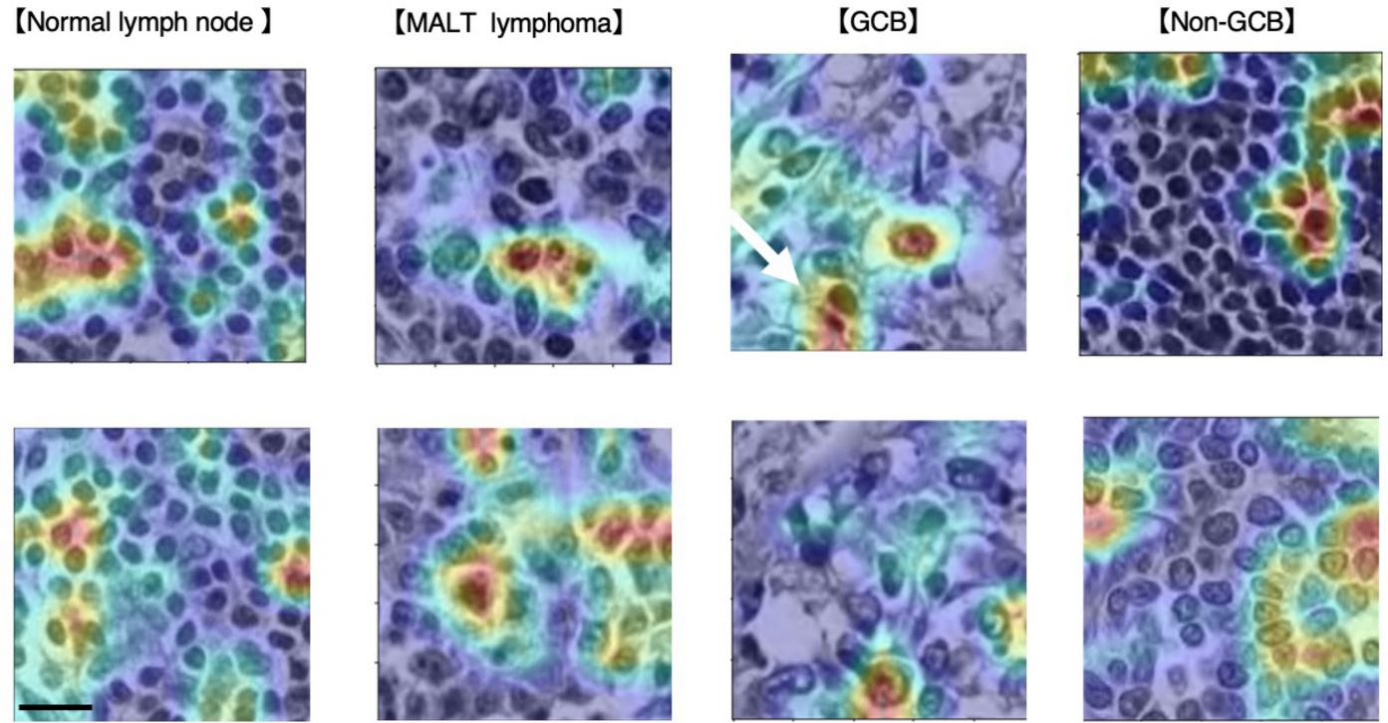
Visualization of data using GRAD-CAM. The image highlights the types of tissues identified. Scale bars represent 20 µm.

The results of the visualization using gradient-weighted class activation mapping (GRAD-CAM) (29, 30) are shown in Fig. 4. The GRAD-CAM was applied to the classification model, which was fine-tuned using EfficientNet. In MALT lymphoma, the focus was primarily on medium-sized lymphoid cells. These morphological characteristics are indeed like MALT lymphoma. For the GCB subtype, the model concentrated on large centroblasts-like tumor cells. Centroblasts are a type of activated B cell found in the germinal center, which is thought to be the origin of the DLBCL GCB type. Morphologically, centroblasts are large cells with coarse chromatin and nuclei containing multiple nucleoli with minimal cytoplasm. It was also noted that in the GCB subtype, there was a tendency to recognize medium-to-large cells other than centroblasts (indicated by white arrows in the upper panel of Fig. 4), which could be a contributing factor to the misclassification. In contrast, for the non-GCB subtype, the focus was on medium to large-sized immunoblastic cells in this study (lower panel in Fig. 4). These observations closely resembled the cells typically scrutinized by pathologists, validating the model's performance (31).

### Classification based on Ki67 positivity

The protein Ki67 serves as a critical tumor marker in IHC and is extensively employed in cancer research and diagnosis (32). The association between Ki67 expression and nuclear chromatin density is well established throughout the cell cycle. Ki67 promotes the phosphorylation of histone H3, a key component of chromatin, and facilitates forming of appropriate chromatin structures during various cell cycle stages (33). High Ki67 expression correlates with fast cancer progression, increased malignancy, and a heightened risk of recurrence, due to active tumor cell division, particularly when its percentage exceeds 40 to 85% (34–37).

We attempted to construct an AI model to predict Ki67 positivity using H&E-stained images of NL, MALT lymphoma, and DLBCL, hypothesizing that chromatin changes are reflected in H&E staining patterns. We adopted 60% (36)—approximately the midpoint of the reported range of 40 (38) to 85% (39)—as the benchmark. In the current study, for the two-class classification, samples with Ki67 values from 0 to 59% were classified as low risk and those with values from 60 to 100% were classified as high risk (37, 40).

**Fig. 5.**
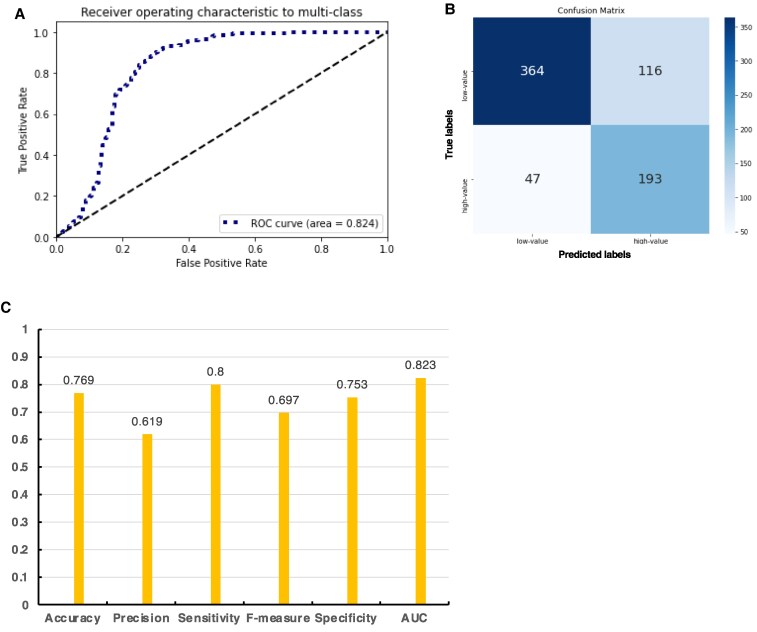
Model performance evaluation in the two-class classification of Ki67 high and low positivity rates. A) An example of ROC curves and B) evaluation metrics. C) Confusion matrix composed of averages from 10-fold cross-validation for low-value Ki67-positive cells (<60%) and high-value Ki67-positive cells (≥60%).

To evaluate the classification performance of the CNN model, test data comprising 240 images from the high-value class and 480 images from low-value class (224 × 224 pixels) were processed (Fig. S5A–D). An example of the ROC curve and confusion matrix for this classification is shown (Fig. 5A and B). In this classification, the high-value class, which has greater clinical relevance, was designated as positive. The results demonstrated that the model accurately identified low- and high-risk classes in over 75% of cases, indicating good classification performance. A specificity value of 0.806 suggested that the model accurately identified the low-value Ki67 class (Fig. 5C and Fig. S5E).

## Discussion

It is entirely feasible for pathologists, as part of their clinical pathology practice, to appropriately select representative images from the whole tissue used for diagnosis and create training images. Moreover, if the image data were created from 250 × 250 pixel photos with a real magnification of 400×, the memory size is about 2 MB. This small image size allows for straightforward data management, making it feasible for clinical laboratories and pathology diagnostic facilities to handle. Thus, implementing a compact CNN model is highly achievable, enabling the development of a system that fully supports pathologists. The EfficientNet model adopted in this study can be easily implemented even in small-scale medical facilities with limited high-performance computing resources, such as GPUs (Graphics Processing Units) (41).

Pretraining with ImageNet drastically reduced the time required for constructing CNNs that cope with a target dataset. We chose ImageNet because we thought that adding a specific cancer type to the training image would not only be biased but would also incur additional costs related to image management. This is the motivation for introducing CNNs pretrained using ImageNet as models for image processing available in the medical field (42–45). For example, such an approach is adopted to develop a model in ultrasound images for computer-aided diagnosis. The VGG16 CNN-based method estimates the osteochondritis dissecans of the humeral capitellum probability of the region of interest (ROI) (43).

It is expected that the approach based on early stopping and cross-validation in the current study can make it possible to avoid overfitting the target dataset. This approach does not always apply to any small dataset, especially for unseen clinical cases with more variability in the members (i.e. images). To examine in detail the limitations of this approach will be beyond the scope of this paper. To apply the proposed approach to other medical datasets remains as a future problem. In (46), we have been engaged in adopting AI technology to medical practices in an ophthalmic surgery, including continuous curvilinear capsulorhexis (CCC), the process of removing the central part of the lens (nuclear extraction), and the process of removing the central part of the lens. A CNN-based method of extracting important phases from videos of cataract surgeries was proposed. A CNN, known as InceptionV3, was employed to construct the model for extraction. The correct response rate of the cataract surgical phase classification was 90.7% for CCC, 94.5% for nuclear extraction, and 97.9% for other phases, with a mean correct response rate of 96.5%. In (47), a method of checking the eye lotion instillation was proposed for ophthalmology patients. It first estimated tilt angles of an eye dropper bottle from acceleration values measured by a triaxial sensor attached to the bottle. Next, we prepared the data to present to the discrimination model, equivalent to a sequence of standardized slope values. It employed a long short-term memory. Once the data to be checked were presented to the model, it produced a certainty degree indicating whether a patient corresponding to the presented data applies eye lotion at the time zone in which a sequence of the tilt values used to prepare the presented data was measured. The final judgment for the instillation depended on thresholding of the certainty degree. Experimental results with practical data demonstrated that the models achieved favorable judgment accuracy for the installation. By analyzing these videos, we have gained experience in the analysis of medical image applying various CNN models. Below, we discuss the pathological significance of our model in more detail.

Our model demonstrated robust functionality in classifying NL and B cell lymphomas, achieving an AUC of over 0.900 across all combinations. For lymphomas with distinctive histomorphological features, such as follicular lymphoma, AI exhibited excellent classification performance (48). On the contrary, although research on diagnosing MALT lymphoma using AI technology is rare (49), as MALT lymphoma is often difficult to distinguish from inflammation, our results indicate that machine learning can effectively diagnose MALT lymphoma (50). High-grade transformation in MALT lymphoma occurs with a likelihood of 5–10%. There is a notably low 2-year survival rate of ∼60% after transformation, highlighting the critical nature of early histological diagnosis for differentiating DLBCL from MALT lymphoma (51, 52). Our model is significant for the differential diagnosis of DLBCL from MALT lymphoma, underscoring the important clinical implications of this research (52). For the subclassification of DLBCL, the advanced gene profiling techniques have been used to observe non-GCB cases with a higher relative contribution of the activation-induced cytidine deaminase-dependent mutational motif RCH (where R = A or G, H = C, T, or A). In GCB cases, a higher relative contribution of the polymerase η-like mutational motif TW (W = A or T) has been observed. These approaches allow the allocation of the tumor cell origin with a high degree of accuracy (precision: 0.86, sensitivity: 0.75, and specificity: 0.95). This classification performance is comparable to the IHC method based on the Hans classification and matches the results of detailed genetic analyses (53, 54). Notably, our model demonstrated that all combinations achieved an AUC exceeding 0.900 in classifying NL and B cell lymphomas, indicating robust capabilities. Specifically, the four-class classification, which includes NL, MALT lymphoma, and DLBCL, achieved an AUC exceeding 0.87, a value comparable to the classification performance achieved through the above genetic analysis (53–55). Additionally, our two-class classification effectively distinguished between the Ki67 low (0–59%) and high (60–100%) classes (AUC = 0.82). Therefore, by combining our three-class classification (AUC > 0.99) or four-class classification models with risk assessment based on Ki67 positivity rates, it is expected to improve prognosis through rapid screening and facilitate the early formulation of treatment plans (56). Finally, we describe the relationship between Ki67 positivity and lymphoma subtypes. Of note, it was significantly different between the MALT and DLBC (GCB, non-GCB) subtypes (*P* = 7.2 × 10^−4^). However, when comparing GCB and non-GCB groups, the Mann–Whitney *U* test yielded a *U* statistic 718.0 and a *P*-value of ∼0.040. Moreover, substantial interindividual variation within the non-GCB group (Supplementary 3E) led to inconclusive findings regarding significant differences in Ki67 positivity rates between these groups. Consequently, differentiating between GCB and non-GCB groups based solely on Ki67 with AI modeling proved challenging, suggesting that predicting high-risk and low-risk classes should be considered independent of DLBCL subtype classification. The application of GRAD-CAM in pathology analysis holds significant potential for visualizing model decision-making processes. However, GRAD-CAM primarily provides qualitative visualizations, which makes it challenging to establish robust, quantitative metrics for clinical decision-making or validation (57). To overcome this limitation, integrating GRAD-CAM with complementary techniques could yield more precise and clinically meaningful insights. Moreover, thorough validation using independent datasets and expert pathological reviews remains crucial.

In this study, we successfully classified low- and high-grade B cell malignancies. Although the model achieved a reasonably high AUC in 160 patient samples, the classification performance is still limited. The expansion of the dataset is essential to address the variability observed in real clinical cases, especially in rare and complex subtypes of malignant lymphomas. In addition, the model can be applied to any type of disease if the pathologist chooses the ROI appropriately.

The main strength of this approach lies in its practicality and compactness. Pathologists can easily integrate the method into their daily work by selecting representative ROIs and using the classification output as a diagnostic reference. These outputs streamline subsequent analyses, such as narrowing down protein staining to essential markers, improving efficiency and specificity. In addition, pathologists can directly validate the AI classification results, incorporate new training images, adjust parameters, and refine the algorithm to improve the model's performance. This iterative process allows for the effective integration of AI into the diagnostic workflow while maintaining confidence in the diagnostic process through direct oversight by pathologists.

It is important to emphasize that AI does not replace physician expertise, but serves as an auxiliary tool to support it (58). The FDA (Food and Drug Administration) (59) mandates that AI-based diagnostic tools that utilize imaging data must be used only by qualified professionals, emphasizing the need for physician oversight. Pathologists can reassure patients that their data will be collected for diagnostic purposes only, will be managed under their direct supervision with robust security measures to protect sensitive medical data, and will adhere to ethical guidelines for the handling of imaging data and associated clinical data. As demonstrated in this study, our model does not require high-performance computing infrastructure and does not impose additional financial burdens on healthcare budgets. This accessibility will eliminate disparities between medical facilities caused by financial constraints and ensure equitable access to high-quality diagnostics, even in complex cases such as lymphoma classification. Similar to the analysis of surgical processes mentioned above (46, 47), our approach is expected to contribute to improving diagnostic standards through the education and training of resident physicians in hospitals.

## Conclusion

Compact and cost-effective AI models offer a practical and scalable solution for integrating AI into the medical field. By aligning with real-world medical needs, such AI systems are expected to play a crucial role in advancing diagnostics and supporting healthcare delivery.

## Materials and methods

### Samples

TMAs containing numerous 1-mL-diameter circular tissue cores were obtained from Biomax (cat. ID LM208, LY800a, LY616a, LY1001d, LY2081, LY2083, LY2084, LY2085, and LM208, US Biomax, Rockville, MD, USA), which included samples of NL, MALT lymphoma, and DLBCL (Fig. 1). These TMAs were reassessed by multiple hematopathologists with over 20 years of experience based on H&E- and IHC-stained sample. Informed consent was obtained from the patients before the primary samples were taken. The Medical Ethics Committee of the Kyoto University Graduate School of Medicine reviewed the research protocol and deemed exempt because the used TMAs were commercially purchased. The profiles of the patients are listed in Table 1.

### Staining and preprocessing

The DLBCL subtype was confirmed by IHC for BCL6, CD10, and MUM1. Staining was performed using the Ventana BenchMark ULTRA (Roche Diagnostics, Basel, Switzerland) with diaminobenzidine chromogen, revealing positive cells with a brown coloration. The antibodies used in this study are listed in Table S2. Image data from 160 individuals comprised 31 GCB, 78 non-GCB, 26 MALT lymphoma, and 25 NL cases (Table 1). Based on IHC data, the preprocessed image data, up to 30 patch images (224 × 224-pixel size), were randomly selected for each patient and labeled GCB or non-GCB.

For the lymphoma subtype classification, the training data included 2,151 patch images for GCB (24 patients), 5,490 for non-GCB (62 patients), 1,842 for MALT lymphoma (21 patients), and 1,350 for NL (20 patients). In addition, data augmentation was performed by including images rotated by 90° and 270°. The test data comprised 210 patch images for GCB (7 patients), 480 for non-GCB (16 patients), 122 for MALT lymphoma (5 patients), and 150 for NL (5 patients).

The Ki67 values were given by Datasheet of TMAs and were labeled for 22 GCB cases (Ki67 value range: 10–100%), 52 non-GCB cases (Ki67 value range: 2–95%), 25 MALT lymphoma cases (Ki67 value range: 1–40%), and 25 NL (Ki67 value range: 0%). The original images were captured using an Olympus OX40 inspection microscope camera at 200 × and 400× magnification. Images were randomly captured from two to eight locations on each tissue section to ensure no overlap in the imaged areas.

### Classification model construction

The process of constructing the lymphoma AI classification model is illustrated in Fig. 2. Initially, pretraining was conducted on EfficientNet-B0 using the ImageNet dataset. Training was implemented using the PyTorch library (https://pytorch.org/). Subsequently, the output layer of the network was modified. The original output layer was set to 1,000 neurons owing to pretraining with ImageNet. After modification, the number of neurons in the output layer was adjusted to match the number of classes (2–4) depending on the targeted lymphoma type. During pretraining with CUReT, the settings included a batch size of 32, the cross-entropy loss function, stochastic gradient descent as the optimization function (momentum: 0.9), and a maximum of 300 epochs. Early stopping was implemented if the validation error increased for ten consecutive epochs, indicating potential overfitting, thereby terminating the training. For image input to the model, the size of the images was resized to 224 × 224 pixels. Data augmentation techniques were applied, including random horizontal flipping and adjustments in brightness, contrast, saturation, hue, and grayscale conversion. In addition, the pixel values were normalized to have a mean of 0 and a SD of 1.

### Adoption of early stopping and cross-validation

To prevent overfitting in the neural networks, we adopted early stopping. Early stopping in our method is based on cross-validation, utilizing K-fold cross-validation to determine the combination of training and validation data.

### Performance comparison verification of CNNs

The number of parameters in the AlexNet, VGG16, ResNet18, SqueezeNet, GoogleNet, and EddicientNet CNN architectures was 57,020,228, 134,276,932, 11,178,564, 724,548, 5,604,004, and 4,012,672, respectively.

## Supplementary Material

pgaf137_Supplementary_Data

## Data Availability

Source data for all figures and supplementary figures are provided with the paper. The code used to analyze the image data can be found at https://github.com/N6atUoH/TumorClassifier. Correspondence and requests for materials should be addressed to T.T. (tsuruyam@ddm.med.kyoto-u.ac.jp).
