# Supplementary material for: AI system for diagnosing mucosa-associated lymphoid tissue lymphoma and diffuse large B cell lymphoma using ImageNet and hematoxylin and eosin–stained specimens
Source: PNAS Nexus. 2025 Apr 30;4(5):pgaf137. doi: 10.1093/pnasnexus/pgaf137 (PMC12069809; doi:10.1093/pnasnexus/pgaf137)
Supplement: pgaf137_Supplementary_Data [file pgaf137_supplementary_data.zip › PNASNEXUS-PNASNEXUS-2024-01062-TR-s01.docx]

Supplementary information

**Artificial Intelligence System for Diagnosing MALT lymphoma and Diffuse Large B cell Lymphoma Using ImageNet and H&E Stained Specimens**

Shuto Yamaguchi^1^, Teijiro Isokawa^1^, Nobuyuki Matsui^1^, Naotake Kamiura^1^*, Tatsuaki Tsuruyama^2,3^*

^1^Department of Electronics and Computer Science, Graduate School of Engineering, Himeji, University of Hyogo, Japan

^2^Department of Drug Discovery Medicine，Graduate School of Medicine, Kyoto University, Kyoto, 606-8315, Japan

^3^Department of Clinical Laboratory, Graduate School of Health Sciences, Kyoto Tachibana University, Kyoto 607-8175, Japan

**Materials and methods**

**The training data for Ki67.** The training data consisted of 5,748 patch images for the low-value class from 69 patients and 2,700 images for the high-value class from 30 patients. Data augmentation was performed by including images of the same size rotated by 90° and 270°. Conversely, the test data included 480 patch images for the low-value class from 17 patients and 240 for the high-value class from eight patients. In both the lymphoma subtype and Ki67 value classification datasets, the ratio of patients in the training to the test data was maintained at 8:2.

**Fine-tuning. Fine-tuning process is depicted in Fig. 2.** Fine-tuning offers the remarkable advantage of reducing the cost required for network training. We used EfficientNet to diagnose lymphoma through fine-tuning. This network adopts a method called "Compound Scaling," which uniformly scales the network’s depth, width, and resolution based on a fixed ratio.

**Training conditions using histopathological images.** We retrained the entire model, including the modified output layer, starting with the parameters obtained from the pre-training. For training purposes, this retraining was performed using data derived from histopathological images. For data augmentation, we applied random horizontal flipping, changing brightness and contrast to adjust the input images. The input images were normalized with a mean of zero and a standard deviation of one. The batch size was set to 32, the loss function used was the Cross-Entropy Loss, and the optimization function was stochastic gradient descent (momentum: 0.9). The maximum number of epochs was set to 100 and *K* = 10, indicating that a 10-fold cross-validation was employed. Early stopping was implemented such that the training ceased if the validation error increased for ten consecutive epochs. The proposed method saves the model parameters at the end of training to complete the network for identifying unknown data. For histological classification, patch images of 224×224 pixels (GCB: 210, non-GCB: 480, MALToma: 122, and NL: 150) were used as test data for evaluating the classification ability of the network model described above.

**Supplementary figures**

**Supplementary Fig.1.** **Model performance evaluation in two-class classification between lymphoma types.** (*A*) An example of ROC curves for comparison and an evaluation metrics for the two-class classification models between NL and DLBCL. (*B*) An example of ROC curve for comparison and an evaluation metrics for the two-class classification models between NL and DLBCL. (*C*) An example of ROC curve for comparison and an evaluation metrics for the two-class classification models between NL and all lymphomas (*n* = 480 images for 16 patients).

**Supplementary Fig.2.** **Model performance evaluation in three-class classifications.** (*A*) A confusion matrice from ten-fold cross-validation, with a thick line indicating the divisions used for metric calculations, distinguishing NL from MALToma and DLBCL. (*B*) An examples of ROC curves from tests on sections comparing NL vs. MALToma vs. DLBCL.

**Supplementary Fig.3.** **Studied CNN models and pre-training databases for four-class classifications.** (*A*) Evaluation metrics for four-class classification among NL, MALToma, GCB, and non-GCB using AlexNet, VGG16, ResNet18, SqueezeNet, GoogleNet, and EfficientNet. (*B*) Studied pre-training databases for four-class classifications. Evaluation metrics for four-class classification among NL, MALToma, GCB, and non-GCB using no pre-training, pre-trained with CUReT, pre-trained with ImageNet, and pre-trained with ImageNet followed by CUReT.

**Supplementary Fig.4.** **CNN models for four-class classifications.** ROC curves (left) from tests on sections and confusion matrices (right). (*A - F*) NL (n =150 images for five patients) vs. MALToma (n = 122 images for five patients) vs. GCB(n = 210 images for seven patients) vs. non-GCB(n = 480 images for 16 patients). (*A*) AlexNet, (*B*) VGG16, (*C*) ResNet18, (*D*) SqueezeNet, (*E*) GoogleNet, and (*F*) EfficientNet. The number of tested image data and patient numbers are also shown in **Table 2**. CNN, convolutional neural network; ROC, receiver operating characteristic.

**Supplementary Fig.5 Profiles of image data for machine learning training and testing.**

The vertical axis represents the number of image data, while the horizontal axis indicates the Ki67 positivity rate. (*A*) Low-value class for training. (*B*) High-value class for training. (*C*) Low-value class for testing. (*D*) High-value class for testing.

**Supplementary Tables**

**Table S1.** Training data and test data set. The first number in the table represents the number of image data, and the number in parentheses indicates the number of patients from whom those images originated.

|  | NL | MALToma | GCB | non-GCB |
| --- | --- | --- | --- | --- |
| Training data | 1350 (20) | 1841 (21) | 2151 (24) | 5490 (62) |
| Test data | 150 (5) | 122 (5) | 210 (7) | 480 (16) |
| Total | 1500 (25) | 1693 (26) | 2361 (31) | 5970 (78) |

NL, normal lymph nodes; MALToma, extranodal marginal zone lymphoma of mucosa-associated lymphoid tissue; GCB, germinal center B-cell; DLBCL, diffuse large B-cell lymphoma.

**Table S2**

Protocol for immunohistochemical assay to detect specific antibodies for DLBCL classification.

| **Antibody** | **Bcl6** | **MUM1** | **CD10** |
| --- | --- | --- | --- |
| Clone | GI191E/A8 | MUM1p | 56C6 |
| Supplier | Roche Diagnostics, Rotkreuz, Switzerland | Roche Diagnostics, Rotkreuz, Switzerland | Roche Diagnostics, Rotkreuz, Switzerland |
| Dilution | ×1 (RTU) | ×50 | ×50 |
| Reaction | 32 min, RT | 30 min, RT | 40 min, RT |
| Antigen Retrieval | Ventana, BenchMark ULTRA, Roche CC1 solution (Std 64 min) | Heat Processor solution pH 9  (40 min) | Ventana, BenchMark ULTRA, Roche CC1 solution (Std 64 min) |
| System | Ultraview | Ultraview | Ultraview |
| Stainer | Roche Ventana (Roche Diagnostics) | Roche Ventana (Roche Diagnostics) | Roche Ventana (Roche Diagnostics) |

RTU, ready to use; RT, room temperature.
