# Supplementary figures and images for: AI system for diagnosing mucosa-associated lymphoid tissue lymphoma and diffuse large B cell lymphoma using ImageNet and hematoxylin and eosin–stained specimens
Source: PNAS Nexus. 2025 Apr 30;4(5):pgaf137. doi: 10.1093/pnasnexus/pgaf137 (PMC12069809; doi:10.1093/pnasnexus/pgaf137)

A

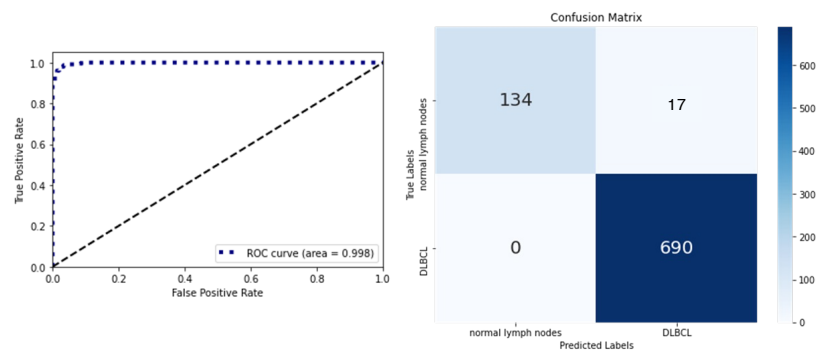

C

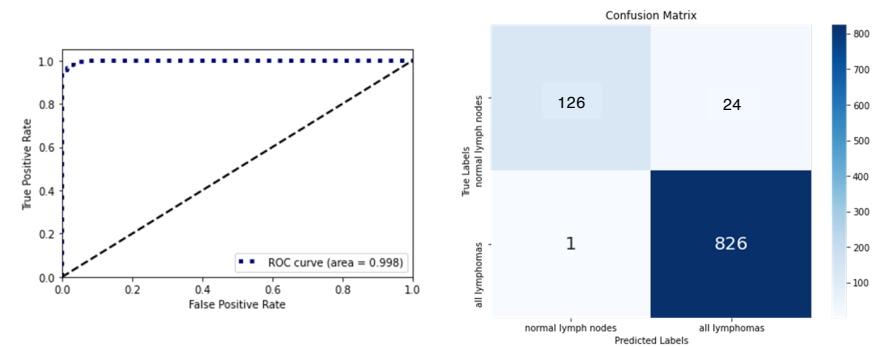

B

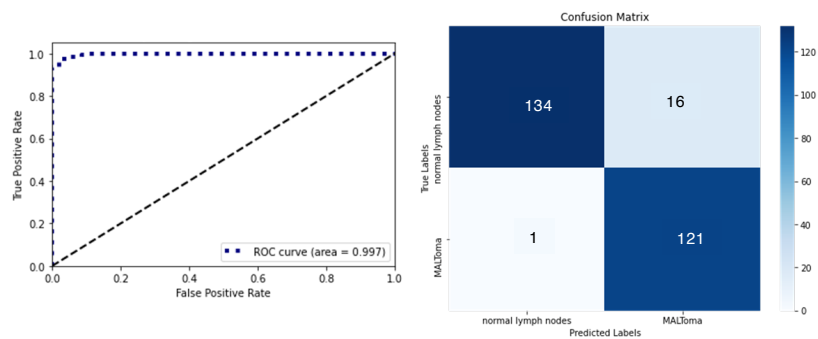

Supplement: pgaf137_Supplementary_Data [file pgaf137_supplementary_data.zip › Supplementary figure 1.pdf]

A

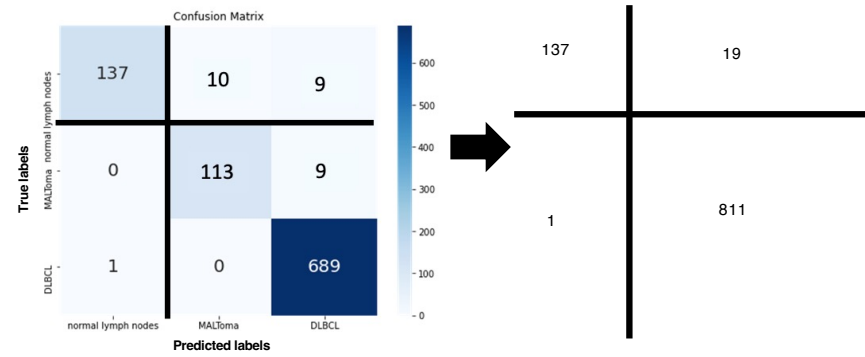

B

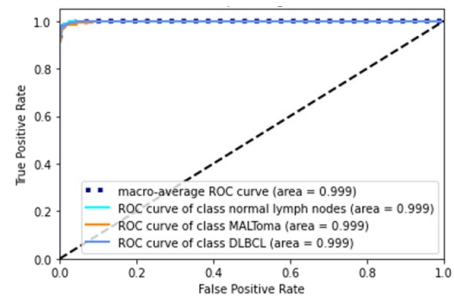

Supplement: pgaf137_Supplementary_Data [file pgaf137_supplementary_data.zip › Supplementary figure 2.pdf]

A

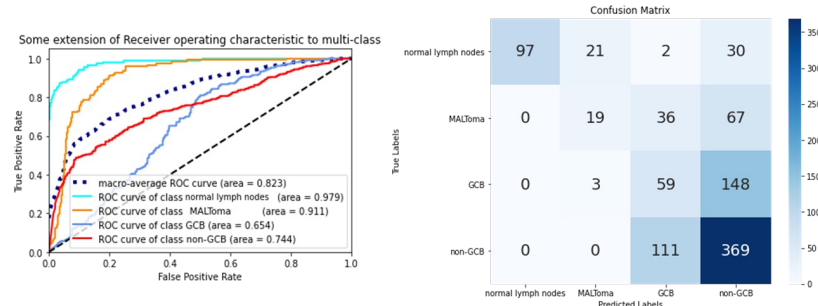

B

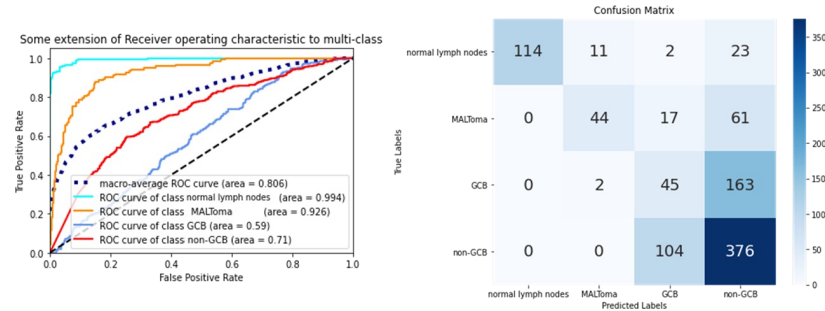

C

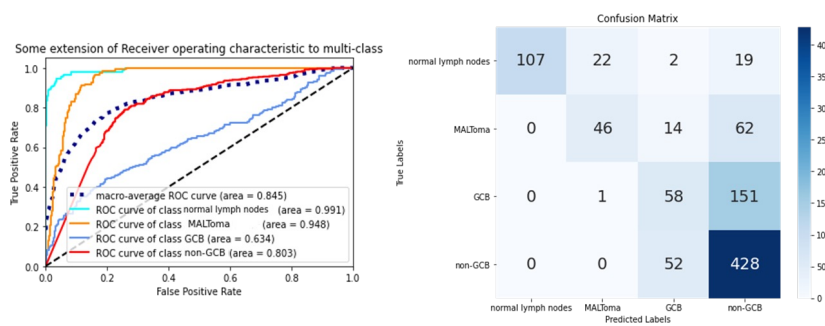

D

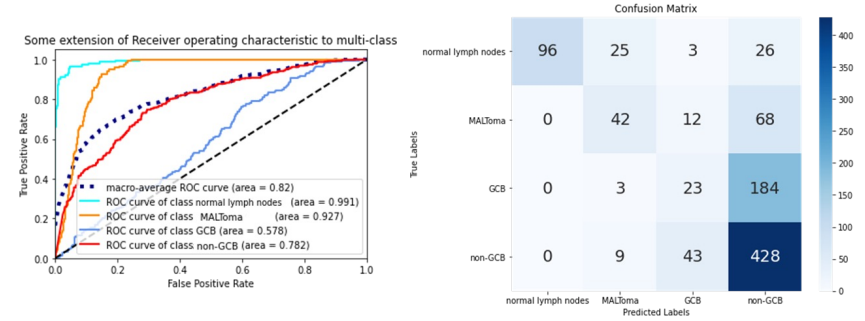

E

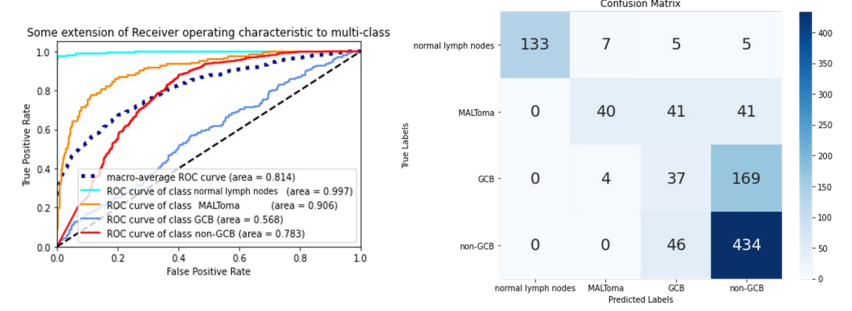

F

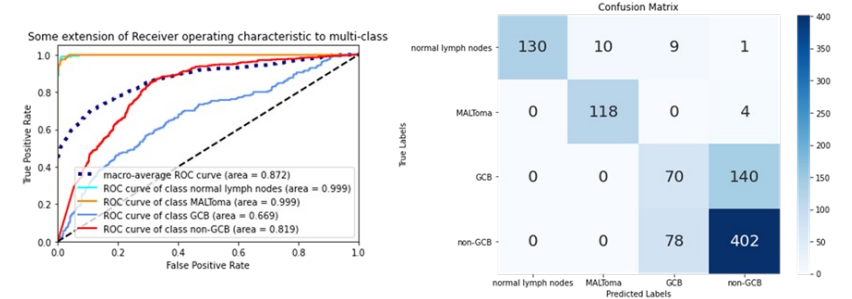

Supplement: pgaf137_Supplementary_Data [file pgaf137_supplementary_data.zip › Supplementary figure 3.pdf]

A

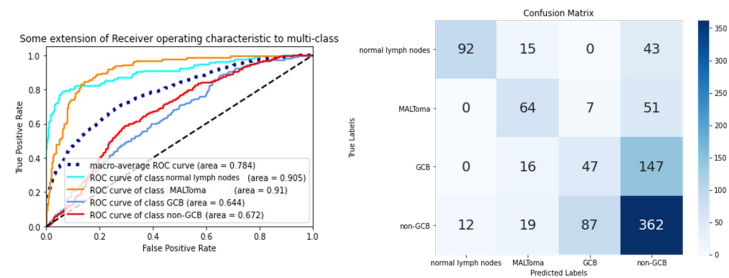

C

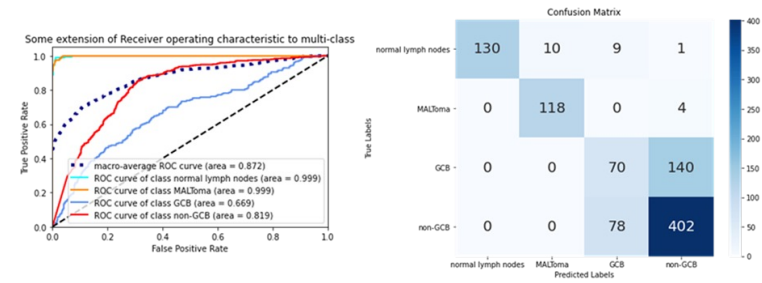

B

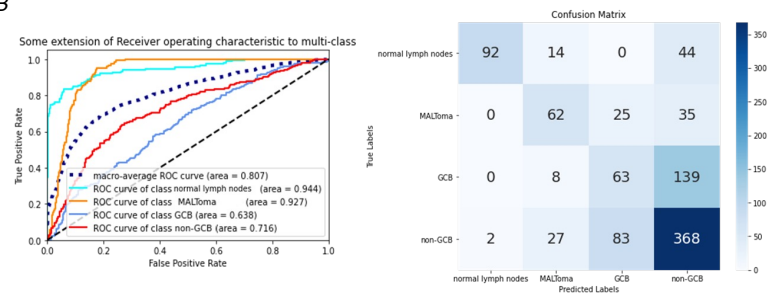

D

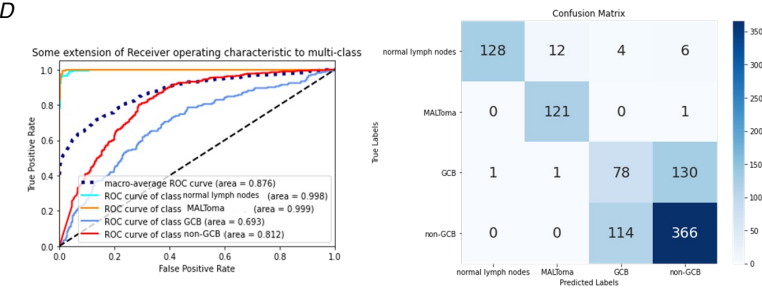

Supplement: pgaf137_Supplementary_Data [file pgaf137_supplementary_data.zip › Supplementary figure 4.pdf]

A

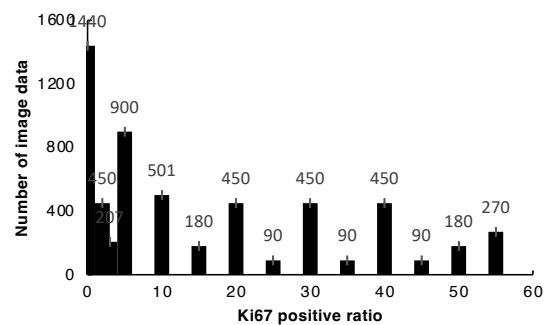

B

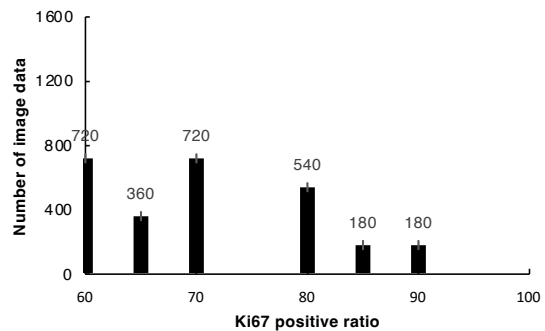

C

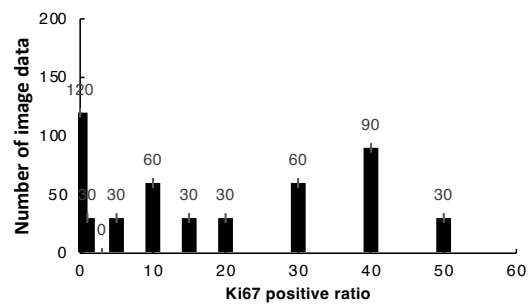

D

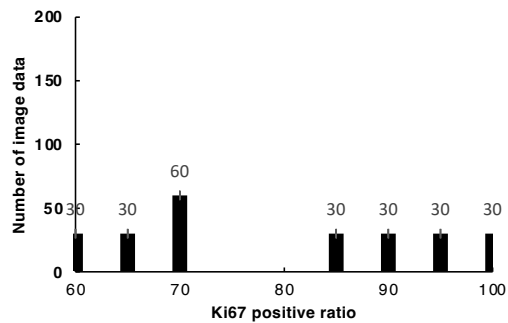

E

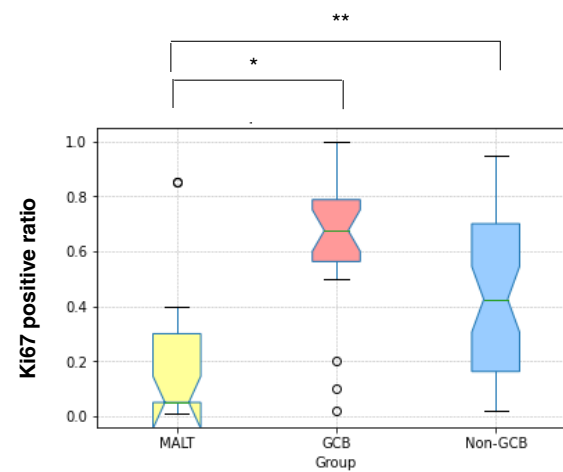

Supplement: pgaf137_Supplementary_Data [file pgaf137_supplementary_data.zip › Supplementary figure 5.pdf]
